# Renal transplant patients’ preference for the supply and delivery of immunosuppressants in Wales: a discrete choice experiment

**DOI:** 10.1186/s12882-017-0720-5

**Published:** 2017-10-02

**Authors:** Anke Hagemi, Catrin Plumpton, Dyfrig A. Hughes

**Affiliations:** 1 Betsi Cadwaladr University Health Board, Ysbyty Gwynedd, Bangor, Wales UK; 2 Centre for Health Economics and Medicines Evaluation, Bangor University, Ardudwy, Holyhead Road, Bangor, Wales LL57 2PZ UK

**Keywords:** Discrete choice experiment, Immunosuppressants, Drug prescribing, Patient preference, Kidney transplantation

## Abstract

**Background:**

Prescribing policy recommendations aimed at moving immunosuppressant prescribing for renal transplant patients from primary to secondary care may result in benefits of increased safety and reduced cost. However, there is little evidence of patients’ preferences for receiving their immunosuppressant therapy from hospitals compared to community dispensing. The aim of this study was to elicit patient preferences for different service configurations focusing in particular on home delivery versus collection of medication from hospital.

**Methods:**

A discrete choice experiment was administered to 265 renal transplant patients in North Wales. Respondents were presented 18 pairwise choices, labelled as either home delivery or hospital collection, and described by the attributes: frequency of supply, waiting time (for delivery or collection) and method of ordering (provider contact, patient contact via phone, patient contact electronically). Data were analysed using a random-effects logit model and marginal rates of substitution calculated based on the waiting time attribute.

**Results:**

A response rate of 63% was achieved, with 5332 usable observations from 150 respondents. Method of delivery (β coefficient 1.21; 95% confidence interval 1.05 to 1.38), frequency of supply (0.05; 0.03 to 0.08) waiting time (−0.00, −0.00 to −0.00), provider contact (desirable) (0.20; 0.12 to 0.27), patient contact by telephone (desirable) (0.09; 0.01 to 0.17) and patient contact electronically (undesirable) (−0.292; −0.37 to −0.21) were statistically significant (*p* < 0.05). Results indicate that patients are willing to increase waiting time by nearly 10 h to have a home delivery service.

**Conclusion:**

Patients indicate a clear preference for a home delivery service. They prefer providers to make contact when new immunosuppressant supplies are required and show preference against ordering medication electronically. A policy for secondary care prescribing and hospital collection of medicines does not align with this preference.

**Electronic supplementary material:**

The online version of this article (10.1186/s12882-017-0720-5) contains supplementary material, which is available to authorized users.

## Background

The safe and effective use of immunosuppressants in the prevention of organ transplant rejection requires careful prescribing, and a high degree of adherence. Adverse events may arise from missed doses [1], medication errors [2], from switching between different brands of the same immunosuppressants [3], or during dispensing. Measures to reduce the likelihood of adverse outcomes, including appropriate prescribing, medicines optimisation strategies and supporting medication adherence, are reinforced in clinical guidelines [4, 5].

Prescribing policies that promote the safer use of immunosuppressants in the UK have centred on increased specialist input from secondary care or tertiary centres to meet the pharmaceutical care needs of patients [6, 7]. Patients may either collect a supply of their medication from the hospital pharmacy following a clinic appointment, or receive a delivery of their immunosuppressants by a registered pharmacy that specialises in home delivery. Patient preferences are important in the context of recommendations that patients should be given a choice in how their medicines are supplied [8]. However, we are aware of only one evaluation of patients’ perspectives of a home delivery service of immunosuppressants [9]. Conducted as a postal questionnaire involving 300 patients at the Oxford Transplant Centre, the study indicated over 98% respondents prefer the home delivery service to the medication supply service previously provided by the hospital. However, the study was limited in terms of methodology through the use of a non-validated questionnaire, incomplete reporting and a risk of social desirability bias that might arise from patients’ reluctance to criticise their health care provider [10].

Discrete choice experiment (DCE) is a quantitative technique for eliciting patients’ stated preferences. It has been applied extensively to assess service users’ preferences for health care service delivery and organization [11], to inform health policy, planning and resource allocation decisions. Within a DCE, respondents are asked to choose between a set of hypothetical but realistic scenarios, which are each described by a number of characteristics (attributes) for which the levels are varied. DCEs assume that respondents’ preference is revealed through their choice decisions [12]. To our knowledge, the only DCEs conducted in renal transplant patients have considered prioritisation of transplant, rather than considering aspects of service delivery [13, 14]. In the context of a policy change, moving prescribing from primary care (where patients obtain their medicines from a community pharmacy) to secondary care involving hospital pharmacies, we aim to elicit patients’ preferences for obtaining their immunosuppressive therapy via home-delivery or by collection from hospital pharmacies.

## Methods

### Setting

The health board in North Wales is responsible for three major hospitals, located in the West, Central and East of the region. The East region is mostly urban or semi-urban, whereas the West and Central areas are more rural with distances from patients’ homes to the nearest hospital being up to 60 miles and requiring up to 2 h of travel time. Current supply of immunosuppressants is via collection alongside other medication prescribed by the general practitioner (GP) on a monthly basis from a local community pharmacy. However the policy recommendation is a change of prescribing responsibility from the GP to hospital (secondary care) based nephrologists. This means that the supply of immunosuppressants has to be arranged by the hospital pharmacies, which can be direct collection from the hospital or provision of home deliveries.

### Study design

A mixed methods approach was taken, which involved qualitative research methods (focus groups) to inform the design of the DCE. Ethics approval was granted by the North Wales Research Ethics Committee (West) reference 11/WA/0244.

### DCE attribute and level selection

Initial attributes and levels of the prescribing service were based on clinical experience with home delivery services of erythropoiesis stimulating agents, a programme which initiated in North Wales in 2007, and a patient satisfaction questionnaire administered to 198 patients in 2008 (response rate 76.8%). Responses to the questionnaire highlighted the importance to patients of the location of medication delivery (home versus hospital versus GP) and identified a prolonged waiting time as a cause of patient dissatisfaction [15]. In the context of this work, waiting time refers to either the length of an allocated delivery time slot for a home delivery, or the time waiting in the hospital pharmacy for collection of the prescription. The time waiting in hospital may be substantially shorter and the collection of medicines from hospital pharmacy usually follows a clinic appointment, however time spent at home may be used more productively. The additional attribute of the interval of ordering was chosen to reflect clinical practice.

Focus groups were convened to discuss the attributes and refine how they were to be presented as part of the DCE, to identify relevant levels, to ensure that they were important and relevant to the patient population and understood by DCE respondents [16]. Twenty patients, randomly sampled from the patient list of 265 patients, were sent an invitation letter outlining the key aims of the study and a consent form should they wish to participate in the focus groups. Nine patients consented to take part in the focus groups and two sessions were facilitated to maximise attendance considering participants’ work commitments and travelling distance to the meeting venue.

The purpose of the first focus group was to assess the relevance and importance of the identified attributes (waiting time, location of collection, interval between supplies, safety or risk of errors, and cost to the National Health Service (NHS) in the UK), and to identify any further attributes using a thematic analysis. The second focus group considered the attribute list and again considered the relevance and importance, with a focus on phraseology, and potential attribute levels.

Focus group participants did not identify any further attributes; however there was concern that the inclusion of a price proxy would result in different interpretation of the meaning of cost (e.g. cost to the patient, drug cost, overall service cost) in the context of healthcare being free at the point of delivery. It was decided, therefore, not to include a price proxy in the DCE. Waiting time was considered as an attribute to estimate DCE respondents’ willingness to give up time for improvements on other attributes. Clinical experience with home delivery of medicines and data on local hospital dispensary waiting times were considered when assigning levels to this attribute. Although the attribute pertaining to prescribing safety/ risk of errors was considered to be important to some focus group participants, it was identified as not suitable for inclusion in the DCE as no meaningful levels could be established as no comparative risk data were available for the different service models. The final selection of attributes and levels used in the DCE are detailed in Table 1.

**Table 1:** Attributes and levels for the discrete choice experiment

Attribute	Definition	Levels
Supply Method	How you collect your supply of immunosuppressant medication	Hospital Supply Home deliveries
Wait	Waiting time on day of tablet collection / delivery	Hospital supply: 10, 20, 60 min Home deliveries: 60, 150, 240 min
Frequency	How often are tablets supplied?	Every month Every 3 months Every 6 months
Ordering	How do I order a new supply of my tablets?	You don’t need to do anything – the provider contacts you when your tablets are ready for collection You order your tablets by phoning the provider You order your tablets from the provider by email or online

### DCE design

A labelled design was used to keep choice sets realistic [17], and to allow for different levels to be assigned to the “waiting time” attribute for the two supply methods.

**Fig. 1:**
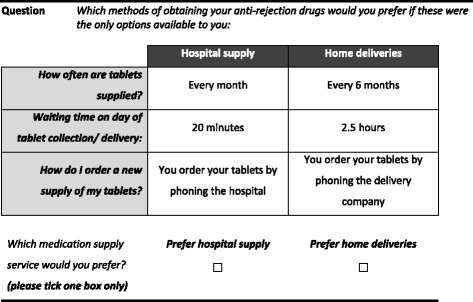
Example of choice set

A full factorial design would result in 54 (3^3^ × 2^1^) profiles and 1431 choice sets, hence a fractional factorial design was used to arrive at a manageable number of choices. The design was based on an orthogonal main effects design taken from a published design catalogue (Design 19a) [18]. Choice options were generated as L^MA^, a labelled experimental design, which allows for the independent estimation of alternative specific attribute effects aiming to increase participants’ familiarity with the context and reduce cognitive burden. The first three columns of the design correspond to attribute levels for choice A, and columns 4–6 of the design correspond to attribute levels for choice B [19]. Choices were presented pair-wise, with respondents being required to make a choice; no “opt out” alternative was presented, as the current situation of GP prescribing will not be continued and non-participation, i.e. not receiving a supply of medication was not considered a valid choice. Figure 1 shows an example of one of the 18 choice sets in the DCE questionnaire. A dominant choice set, sometimes used to test for validity of responses, was not included as respondents’ preferred levels for each attribute were unknown *a priori*.

Information on respondents’ characteristics were collected to test the hypotheses that: (i) previous experience with home deliveries might result in preconceptions about the new service; (ii) travelling distance to the hospital clinic may influence a patient’s willingness to collect their medication from hospital; (iii) patients in full time employment may find home deliveries more inconvenient; (iv) access to a computer may facilitate medication ordering by email or online; and (v) patients may have different preferences depending on their region of residence, which determines the serving nephrology centre. Responses to other supplementary questions informed an assessment of the feasibility of providing a secondary care based prescribing service (e.g. establishing the risk of waste as a result of frequent changes to drug treatment or dosing).

Considerate of the local population, the questionnaire was presented in a bilingual format (English and Welsh).

### Pilot

Patients who attended either focus group meeting were invited to return for a second meeting to review the questionnaire design. Participants were asked to complete and comment on various versions on the DCE questionnaire and the participant invitation letter, aiming to ensure that the DCE task was clearly presented and not overly burdensome. To facilitate understanding and consistency in respondents’ choices, a clearly explained example of a choice set was presented on the first page of the questionnaire.

### Recruitment

Questionnaires were mailed to all transplant patients under the care of the three nephrology centres of the Betsi Cadwaladr University Health Board which serves the North Wales population of 678,000 people. A pre-paid return envelope was included. Where necessary, one reminder was sent out 2 weeks after the initial distribution of the questionnaires. Consent to participate was assumed with the return of completed questionnaires.

### Data analysis

The DCE was analysed using a random effects logit model in STATA® Version 13 (Statacorp, TX), with choice of delivery method specified as the dependent variable. The home-delivery or hospital collection label was entered as the alternate specific constant. Effects coding was applied for categorical variables to ensure that preference statements could be interpreted independent of the current state [20]. The significance, sign and relative magnitude of the regression coefficients were used to estimate the importance of attributes. A positive coefficient indicates that higher levels of the attribute are preferred. Trade-offs among attributes were estimated by marginal rates of substitution (MRS), calculated as the ratio of coefficients of one attribute relative to the coefficient for waiting time. From this, the amount of extra time which a patient is willing to wait for different levels of other attributes can be inferred.

Utilities of home delivery and hospital supply were calculated by weighting the results of the regression against potential outcomes using the formula

$$ Utility={\beta_{Delivery}}^{\ast}\mathrm{Delivery}+{\beta_{Freq}}^{\ast}\mathrm{Frequency}+{\beta_{Wait}}^{\ast}\mathrm{Wait}+{\beta_{Provider}}^{\ast}\mathrm{Provider}+{\beta_{Phone}}^{\ast}\mathrm{Phone}+{\beta_{Electronic}}^{\ast}\mathrm{Electronic} $$

We assumed home deliveries would be made on a 3-monthly basis with a typical wait of 4 h; whilst for hospital supply, we assumed a 20 min waiting time, 3-monthly delivery, and telephone contact for re-supply.

Confidence intervals for coefficients and MRS were calculated using a non-parametric bootstrap approach. Subgroup analyses were performed to aid further interpretation and generalisability of the results. These were defined *a priori* according to patients’ experience of home delivery; region; distance to clinic; employment status and type of transport. A subgroup was considered valid for analysis if it included 50 or more patients; we considered smaller samples to lack statistical powering. Subgroup models were compared with the base-case model for goodness of fit using a log-likelihood ratio test. The potential for false positive results from multiple comparisons required a Bonferroni correction which reduced the *p*-value for 95% significance to *p* = 0.0125.

## Results

All 265 renal transplant patients across North Wales were invited to participate. Of these, 166 questionnaires were returned, resulting in an overall response rate of 63%. The response rate varied by region of nephrology centre: 55.8% for east, 62.5% for central and 72.3% for west. Patient characteristics are summarised in Table 2.

**Table 2:** Patient characteristics

Characteristic	
Age [years], n (%), *N* = 166	
18 to 30	3 (1.8)
31 to 50	48 (28.9)
51 to 70	83 (50.0)
Over 70	32 (19.3)
Gender, n (%), N = 166	
Male	108 (65.1)
Betsi Cadwaladr University Health Board Region, n (%), *N* = 164	
East	72 (43.9)
Central	45 (27.4)
West	47 (28.7)
Current supply of immunosuppressants, n (%), *N* = 162	
GP and community pharmacy	125 (77.2)
GP (dispensing practice)	19 (11.7)
Hospital	18 (11.1)
Transport to clinic, n (%), *N* = 151	
Car	128 (84.8)
Public transport	15 (9.9)
Taxi	0 (0)
Hospital transport	8 (5.3)
Walk	0 (0)
Other	0 (0)
Distance to local transplant clinic [miles], n (%), *N* = 156	
< 20	111 (71.2)
≥ 20	45 (28.8)
Travelling time local transplant clinic [min], mean (SD), *N* = 147	34 (32.0)
< 30	84 (57.1)
≥ 30	63 (42.9)
Experience with home delivery, n (%), N = 162	
Yes	69 (42.6)
No	93 (57.4)
Employment status, n (%), *N* = 159	
Full time work	42 (26.4)
Part time work	20 (12.6)
Not in employment/ retired	97 (61.0)
Access to the internet at home, n (%), N = 159	
Yes	115 (72.3)
No	44 (27.7)
Last change to immunosuppressant medication, n (%), *N* = 163	
In previous 1 month	17 (10.4)
In previous 1–3 months	14 (8.6)
In previous 3–6 months	16 (9.8)
More than 6 months	116 (71.2)

The number of patients (N) varies due to missing data

Seven DCE responses were excluded as respondents’ annotations of the questionnaire clearly indicated a limited understanding of the DCE methodology. Nine further responses were excluded due to a small number (6 or less) of completed choice sets. 133 respondents completed all 18 choice sets. 76 respondents (51%) exhibited evidence of a dominant preference towards either home deliveries (*n* = 55) or hospital supply (*n* = 21), that is, they were non-traders. These were included in the base case analysis, resulting in 5332 observations from 150 patients.

### Base case results

All attributes were statistically significant (*p* < 0.05). The directions of coefficients are consistent with expectations, where hypothesised *a priori*. For example the negative coefficient (β_Wait_ = −0.0021) for waiting time indicates a preference towards a shorter wait for medication supply. Table 3 summarises the results of the base case model.

**Table 3:** Discrete choice modelling results

Attribute	β-coefficient (95% confidence interval)	*P*-value	Marginal rate of substitution (95% confidence interval)
Home deliveries	1.21 (1.05, 1.38)	0.000	588 (422, 1010)
Frequency	0.05 (0.03, 0.08)	0.000	26 (12, 54)
Wait	−0.00 (−0.00, −0.00)	0.000	n/a
Ordering_provider	0.20 (0.12, 0.27)		96 (50, 196)
_ phone	0.09 (0.01, 0.17)	0.026	43 (5, 110)
_ electronic	−0.29 (−0.37, −0.21)	0.000	−139 (−276, −85)
Constant	−0.61 (−0.75, 0.50)	0.000	n/a

The absolute values of coefficients indicate their relative importance on patients’ choice. Method of delivery had the greatest absolute value of the coefficient (β_Delivery_ = 1.21, 95% CI 1.05 to 1.39), followed by the methods patient order their medication supply. The frequency of supply (β_Freq_ = 0.05, 95% CI 0.03 to 0.08) and a unit change in waiting time [minutes] (β_Wait_ = −0.00, 95% CI −0.00 to −0.00) were the least important attributes. Patients were more likely to choose infrequent delivery of medicines and initial contact for ordering made by the health care provider. Increased waiting time and the need for patients to initiate the medication ordering process themselves –either by phone or online– decreased the probability of patients choosing a method of medication delivery.

Based on calculations of marginal rates of substitution (MRS), the results indicate that patients will accept an additional 26 min (95% CI, 12 to 54 min) of waiting time if the interval between medication supplies was increased by one month. Home delivery supplies were valued at almost 10 h of waiting time. A reduction in waiting time of over 2 h would be required for patients to accept online ordering of their medications.

Total utility for home delivery was 0.458 (95% CI 0.316 to 0.601). Hospital supply yielded a significantly lower utility of −0.410 (95% CI −0.514 to −0.302).

### Subgroup analysis

Four subgroups satisfied the criteria for analysis: region (west and central versus east), distance to clinic, experience with home deliveries, and employment status. All subgroups were shown to be significant in terms of model fit, compared with the base case (*p* < 0.01).

Patients served by the central and west (more rural) regional nephrology centres within the Health Board show a higher preference to home deliveries than patients in the east, with a willingness to wait an extra 711 min (more than 11¾ hours) and 444 min (almost 7½ hours) for home delivery, respectively. The travel attribute was not significant for patients living within 30 min of their local hospital clinic; but for those living 30 min or more away, were willing to wait an extra 347 min (5¾ hours) for home delivery.

Patients with previous experience of home deliveries show a weaker preference for home delivery supply compared to those who have not received medication deliveries to their homes. They were willing to increase waiting time by 531 min (nearly 9 h) compared to 623 min (over 10 h), respectively. Of all subgroups, patients in full time employment showed the lowest preference for home deliveries and willing to increase waiting time by only 271 min (4½ hours) for this service.

Marginal rates of substitution (Additional file 1) for the subgroup analysis showed no significant differences in trading, either among subgroups, or compared to the base case.

## Discussion

The present study reports on preferences of renal transplant patients in North Wales for the method of obtaining supplies of immunosuppressive therapy. We found the method of delivery to have, by far, the greatest impact on patients’ preference, with home deliveries identified as the preferred option. Respondents were willing to increase wait time by nearly 10 h for home delivery, and patients’ utility, based on a typical home delivery service, was significantly higher than for collection at hospital pharmacies. The least desired attribute was electronic ordering, with respondents willing to increase waiting time by over 2 h extra to avoid this option. This result should be in the context of 28% of respondents not having access to the internet at home.

Patients were more likely to choose a delivery method if the interval between deliveries was high and the initial contact for ordering a supply was made by the health care provider. Implementing a service addressing both these attributes may however significantly increase the cost of providing the service: more staffing time is required for actively contacting patients compared to responding to requests for a medication supply. Increased intervals between deliveries may result in medication wastage due to dose changes or expired medication if stocks are not rotated following the receipt of a new supply. However, in the sampled population, the risk of medication wastage due to dose changes is low as most patients were on stable doses of immunosuppressants.

Analysis of marginal rates of substitutions within the subgroups “region” and “distance to hospital” supports the hypothesis that patients living further away from the base hospital in rural settings have a stronger preference for home deliveries compared to patients in an urban setting. No published studies have been identified that explored the influence of rurality on patient’s preference on the method of medication supply in transplant patients or other patient groups. Our findings add to other known characteristics of transplant patients who do not live in close proximity to a hospital. Research from the USA, for instance, indicates that distance to a transplant centre may reduce transplant waiting list registration rate and transplant rate [21, 22] while patients living in rural areas may exhibit reduced adherence to immunosuppressant therapy [23] compared to patient in urban areas. No similar studies on the possible impact of rurality on renal transplant patients have been published in the UK [24].

The results of this study are generalizable to other regions of Wales and to other comparable regions of the UK. Despite the administration of the NHS having been devolved to individual countries within the UK, the delivery of pharmacy services for transplant patients is modelled on the same options as considered in our DCE. While North Wales is sparsely populated in comparison to many urban locations in the UK, our sub-group analysis allowed for exploration of differences between urban and rural regions, and indicated a higher preference for pharmacy collection in populated areas which are closer to hospitals. Many countries operate a home delivery service, and our analysis of patients with prior experience of this indicates that they would prefer not to wait as long for their medicines compared to patients with no prior experience. This may indicate a revealed preference of some dissatisfaction with home delivery service, though overall, this was still greatly preferred over hospital pharmacy collection. In common with other DCEs, our analysis is restricted in its generalisability to healthcare systems and settings where the choice of attributes and their associated levels are applicable, and which are represented by our sample population. As such, the findings may have limited generalisability to patients, payers, healthcare systems or jurisdictions beyond the UK.

The study benefited from an acceptable response rate and a high proportion of questionnaires completed in full. We used a rigorous and robust choice-based format to elicit patients’ stated preferences, and a systematic approach to identify relevant attributes and assign appropriate levels.

There are a few caveats to the study, however, including responder bias arising from the familiarity of patients in the west region with the lead researcher, and opt-out being offered while patients were in possession of the questionnaires (those who did not participate might not have done so because they had first read through the questionnaire). A second potential limitation was the use of a forced choice format which, while being appropriate to the policy context, does not allow for patients to indicate that they did not prefer one supply method over the other. Inclusion of an option of “no preference”, however, would have impacted on the number of discrete choices to the extent that response rate might have been adversely affected. Labelling the choice sets with the two possible supply methods represented a third limitation as this led to a significant number of patients exhibiting a dominant preference by choosing home deliveries irrespective of the levels of other attributes in the choice set. We also note that when analysing data on waiting time, no distinction was made between time waiting at the hospital versus time waiting at home, and no account was made for travel time to the hospital, however given that patients collect their prescriptions following an appointment, this is not an additional expense. No attributes describing adherence to treatment or medication safety were included in this DCE. While there is no difference in the frequency of clinic appointments with either method of medication delivery, collection of medicines at hospital pharmacies might provide an opportunity for patient counselling, including matters relating to adherence. Finally, a transcription error meant that 2 of the 18 choice sets did not correspond to the original design; but extensive testing for level balance and orthogonality confirmed that neither were compromised nor affected the validity of the results.

Waiting times are often used as a key performance indicator to measure workload and performance in pharmacy outpatient medication supplies. However, this study showed that patients are willing to accept a significant increase in waiting time for other preferred attributes describing a medication supply service. This should be considered by policy decision makers when evaluating services affecting medication supply. Recent changes to commissioning of solid organ transplantation by NHS England (2014) will result in a significant shift of immunosuppressant prescribing from primary care to transplant centres or secondary care providers with assumed benefits on prescribing safety and cost [7]. The implication of a strong preference for home delivery service, particularly for patients more distant from specialist renal centres, ought to be considered in future commissioning of pharmaceutical services.

## Conclusions

These first insights into the preferences of renal transplant patients suggest that patients have a preference for home-delivery of their immunosuppressant medications. The strength of this preference is increased for patients who live in more rural areas, but decreases in patients who are in full time employment. These findings and influencing factors should be considered in arriving at policies aimed to maximise adherence and patient safety.

## Additional file

Additional file 1: Marginal rates of substitution for the subgroup analysis. (DOCX 18 kb)
