# Supplementary material for: Renal transplant patients’ preference for the supply and delivery of immunosuppressants in Wales: a discrete choice experiment
Source: BMC Nephrol. 2017 Oct 2;18:305. doi: 10.1186/s12882-017-0720-5 (PMC5625806; doi:10.1186/s12882-017-0720-5)
Supplement: Additional file 1: — Marginal rates of substitution for the subgroup analysis. (DOCX 18 kb) [file 12882_2017_720_MOESM1_ESM.docx]

**Additional file 1**: Marginal rates of substitution for the subgroup analysis.

|  |  |  |  |  | Marginal rate of substitution (mins) with respect to waiting time (β_attribute_/ β_time_) | | | | | | | | | | | | | | | LL* |
| --- | --- | --- | --- | --- | --- | --- | --- | --- | --- | --- | --- | --- | --- | --- | --- | --- | --- | --- | --- | --- |
| Subgroup | | β_time_ | 95% CI | | Home | 95% CI | | Freq. | 95% CI | | Provider | 95% CI | | Phone | 95% CI | | Online | 95% CI | |  |
| Base case | | -0.0021 | -0.0031 | -0.0010 | 588 | 422 | 2009 | 26 | 12 | 54 | 96 | 50 | 196 | 43 | 5 | 110 | -139 | -276 | -85 | -3522 |
| Experience of home delivery | | | | |  |  |  |  |  |  |  |  |  |  |  |  |  |  |  |  |
|  | Yes | -0.0019 | -0.0034 | -0.0004 | 531 | 321 | 1910 | 13 | -18 | 79 | 121 | 39 | 544 | 47 | -37 | 205 | -168 | -701 | -67 | -1519 |
|  | No | -0.0022 | -0.0037 | -0.0010 | 623 | 423 | 1255 | 34 | 16 | 84 | 81 | 32 | 195 | 40 | -8 | 124 | -122 | -279 | -61 | -1996 |
| Region | |  |  |  |  |  |  |  |  |  |  |  |  |  |  |  |  |  |  |  |
|  | West & Central | -0.0020 | -0.0034 | -0.0005 | 711 | 458 | 2168 | 28 | 8 | 98 | 108 | 40 | 415 | 77 | 19 | 269 | -184 | -650 | -96 | -1876 |
|  | East | -0.0021 | -0.0036 | -0.0054 | 444 | 288 | 1283 | 23 | 3 | 99 | 79 | 18 | 292 | 6 | -69 | 96 | -85 | -363 | -20 | -1595 |
| Distance to clinic (minutes) | | |  |  |  |  |  |  |  |  |  |  |  |  |  |  |  |  |  |  |
|  | ≤ 30 | -0.0015 | -0.0025 | 0.0009 | 1735 | -19881 | 11767 | 71 | -833 | 591 | 152 | -2017 | 1195 | 148 | -1727 | 1303 | -300 | -2491 | 3614 | -1322 |
|  | > 30 | -0.0028 | -0.0040 | -0.0014 | 347 | 264 | 568 | 17 | 4 | 40 | 85 | 45 | 168 | 21 | -17 | 67 | -106 | -209 | -62 | -2170 |
| Full time employment | | |  |  |  |  |  |  |  |  |  |  |  |  |  |  |  |  |  |  |
|  | Yes | -0.0023 | -0.0039 | -0.0002 | 271 | 159 | 961 | 57 | 23 | 306 | 9 | -116 | 144 | -18 | -211 | 82 | 9 | -103 | 188 | -1056 |
|  | No | -0.0021 | -0.0033 | -0.0008 | 719 | 490 | 1592 | 12 | -4 | 38 | 135 | 73 | 322 | 70 | 20 | 201 | -205 | -526 | -120 | -2421 |

* Log-likelihood
